# Tapping into a new hand‐motor biomarker of the dementia continuum ‐ integration and validation in a cognitive clinic

**DOI:** 10.1002/alz.088451

**Published:** 2025-01-09

**Authors:** Xinyi Wang, Aidan Bindoff, Rebecca J St George, Katherine Lawler, Alastair J Noyce, Quan Bai, Son Tran, Katharine Salmon, James C Vickers, Jane E Alty

**Affiliations:** ^1^ Wicking Dementia Research and Education Centre, University of Tasmania, Hobart, TAS Australia; ^2^ School of Psychological Sciences, University of Tasmania, Hobart, TAS Australia; ^3^ La Trobe University, Bundoora, VIC Australia; ^4^ Queen Mary University of London, London, England UK; ^5^ School of Information and Communication Technology, University of Tasmania, Hobart, TAS Australia; ^6^ Royal Hobart Hospital, Hobart, TAS Australia; ^7^ School of Medicine, University of Tasmania, Hobart, TAS Australia

## Abstract

**Background:**

The process for assessing cognitive symptoms in clinic is time consuming, expensive and usually requires multiple clinicians. There is an urgent need for accessible, quick and low‐cost methods to aid identification of people at the early stages of the dementia continuum. Emerging evidence shows that simple hand movement tests could aid risk stratification. We evaluated the classification accuracy of the new online TAS Test alternate tapping test in the ISLAND Cognitive Clinic in Tasmania, Australia, for dementia, mild cognitive impairment (MCI) and subjective cognitive decline (SCD) from control subjects.

**Method:**

212 participants (69.0 ± 9.3 years old) were recruited: 130 from the Clinic (50 diagnosed with dementia, 54 with MCI, 26 with SCD) and 82 cognitively healthy controls. All Clinic participants had a gold‐standard interdisciplinary consensus diagnosis after detailed neuropsychological tests and completed a 60‐second alternate key tapping test on the TAS Test website during their standard clinic visit. Healthy controls completed the Addenbrookes Cognitive Examination with a score of 95 or higher and TAS Test in clinic. Frequency, variability, key press duration and accuracy scores were calculated. Regression models comprising all motor features, adjusted for age and sex, were compared to their respective null models, comprising age and sex, with area under ROC curves (AUC).

**Result:**

Compared to the baseline model, tapping motor data improved classification of people with dementia (AUC = 0.89, 95% CI = [0.84,0.95], p=0.025), MCI (AUC = 0.8, 95% CI = [0.73,0.87], p=0.002) and SCD (AUC = 0.75, 95% CI = [0.63,0.86], p = 0.004) from cognitively healthy controls. The motor features also helped distinguish dementia (AUC = 0.9, 95% CI = [0.84,0.97], p = 0.024) and MCI (AUC = 0.79, 95% CI = [0.67,0.91], p = 0.022) from SCD, but not dementia from MCI (p = 0.221).

**Conclusion:**

A 60‐second simple keyboard tapping test helped distinguish people with SCD, MCI, or dementia from cognitively healthy controls and holds potential as a new low‐cost and clinically‐accessible motor biomarker.

